# Subtyping colorectal cancer based on septic shock-associated genes: prognosis and immune characteristics

**DOI:** 10.3389/fgene.2024.1468424

**Published:** 2024-11-15

**Authors:** Jinkai Zhao, Jiaan Chen, Jiancheng Zhang, Xuming Pan, Buhai Xu, Jinli Miao, Wenmin Wang, Guangjun Jin

**Affiliations:** ^1^ The Second School of Clinical Medicine, Zhejiang Chinese Medical University, Hangzhou, Zhejiang, China; ^2^ Department of Emergency, The Second Affiliated Hospital of Zhejiang Chinese Medical University, Hangzhou, Zhejiang, China; ^3^ The Yangtze River Delta Biological Medicine Research and Development Center of Zhejiang Province, Yangtze Delta Region Institution of Tsinghua University, Hangzhou, Zhejiang, China

**Keywords:** clustering, colorectal cancer, immune, septic shock, sepsis

## Abstract

**Background:**

Sepsis and colorectal cancer (CRC) are leading causes of death. Given their mutual dependence for susceptibility, we used bioinformatics to explore potential connections between septic shock (SS) and CRC.

**Methods:**

We identified 452 co-expressed genes between SS-related differential expression genes (SS-DEGs) and CRC patient-expressed genes (TCGA-CRC genes). CRC samples were categorized into two cluster subgroups through hierarchical clustering. We then compared the prognosis and immune landscapes of the two cluster subgroups through survival analysis, immune microenvironment analysis, and immune therapy response evaluation.

**Results:**

Clustering analysis of the 452 CRC patient-expressed SS-DEGs identified two subtypes: SS-like CRC (SL-CRC) and non-SS-like CRC (NSL-CRC). There were no significant differences in overall survival between the CRC subtypes. However, the subtypes displayed significant differences in immune score, stromal score, and ESTIMATE score. Based on immune therapy databases, there were also significant differences in responses to anti-CTLA-4 and anti-PD-1 immune checkpoint inhibitors between the subtypes.

**Conclusion:**

Our study reveals significant differences in the immune microenvironment and immune therapy responses between SL-CRC and NSL-CRC subtypes. These findings provide a foundation for identifying new therapeutic targets and developing personalized treatment strategies for specific CRC subtypes.

## Introduction

Sepsis is a complication resulting from a host’s dysregulated response to a primary infection, often leading to organ dysfunction or death (Singer et al., 2016). Despite advancements in medical care, sepsis still reported 48.9 million cases globally in 2017, with 11 million associated deaths (Rudd et al., 2020). Septic shock (SS), a severe form of sepsis, poses a higher risk of mortality (Das, 2023). Colorectal cancer (CRC) affects the colon or rectum and is the third most common cancer worldwide, as well as the second leading cause of cancer-related deaths. In 2020, there were over 1.9 million new cases of CRC and more than 930,000 deaths (The World Health Organization, 2024). Sepsis is a common complication in cancer, significantly associated with increased risk in several cancers, including CRC (Liu et al., 2019; Rhee et al., 2017). Postoperative sepsis complications negatively affect the prognosis of CRC (Levy et al., 2017). Inflammatory and septic infections may also present early signs of CRC (Hotchkiss et al., 2013; Sidhu et al., 2019). The mutual dependence for susceptibility of sepsis and CRC suggests common biological traits between them (Tripathi et al., 2020).

Molecular mechanisms in sepsis and CRC might share intersecting biological pathways, with immune and inflammatory responses playing crucial roles in both diseases. Unlike earlier concepts, excessive inflammatory responses and immune suppression occur successively. Immune suppression can occur simultaneously with excessive inflammation, particularly in viral-induced sepsis (Fu et al., 2023; Hotchkiss et al., 2013). Additionally, studies have shown that patients with sepsis and those with cancer exhibit similar immunosuppressive responses. Both conditions are characterized by reduced expression of MHC molecules, diminished production of IFN-γ by T cells, enhanced signatures of myeloid-derived suppressor cells and regulatory T cells, and increased levels of inhibitory receptor ligands (Hotchkiss and Moldawer, 2014; Washburn et al., 2019). Inflammation in CRC can be categorized into three types: chronic inflammation before tumor therapy, tumor-induced inflammation, and treatment-induced inflammation, all promoting activation of innate immune cells and establishment of an immunosuppressive tumor microenvironment (Schmitt and Greten, 2021).

Research on the role and clinical significance of SS-related genes in CRC is still relatively limited. Therefore, we utilized bioinformatics to explore the potential relationship between SS-related differential expression genes (SS-DEGs) and CRC. In our study, we collected SS-DEGs from the published literature and extracted 452 SS-DEGs expressed in CRC patients. Using hierarchical clustering, we divided the CRC samples into two clusters, categorizing them into SS-like CRC subgroups (SL-CRC) and non-SS-like CRC subgroups (NSL-CRC). We conducted survival analysis, immune microenvironment analysis, and immune therapy response evaluation to investigate differences in prognosis, immune microenvironment, and immune therapy response between the two clusters.

## Materials and methods

### Data collection

TCGA (The Cancer Genome Atlas, https://www.cancer.gov/tcga) data were used to obtain gene expression data for rectal adenocarcinoma (TCGA-READ) samples and colon adenocarcinoma (TCGA-COAD) samples as TCGA-CRC samples. We excluded samples that were unqualified due to poor sequencing results from formalin-fixed paraffin-embedded tissues (n = 19), as well as samples from normal tissues (n = 51) and those lacking clinical data (n = 30), resulting in a total of 598 samples comprising the TCGA-CRC dataset. Data for SS were obtained from the Gene Expression Omnibus (GEO) database (https://www.ncbi.nlm.nih.gov/geo/), under GEO accession: GSE26440, which includes 32 healthy control samples and 98 SS samples (Wong et al., 2009). In cases where expression data contained missing values, genes with these missing values were excluded.

### Identification of SS-DEGs and hierarchical clustering of CRC

We preprocessed the TCGA-CRC and GSE26440 data using R (version 4.3.0), standardizing across samples through Z-score normalization. Batch effects between different sample batches were removed using the removeBatchEffect function from the ‘limma’ package (version 3.56.2). Differential analysis on the GSE26440 dataset was also conducted using the ‘limma’ package, with an FDR <0.05 and |log2FC| > 1 as the threshold for selecting SS DEGs (Bioinformatics for Biologists, 2020; Love et al., 2014).

$$\log2\left(FC\right)=\log2\left(\frac{{Expression\;\;Level}_{Experimental}}{{Expression\;\;Level}_{Control}}\right)$$

$$FDR=\frac{Expected\;False\;Positives}{Total\;Significant\;Results}$$

Expression data for SS-DEGs were then extracted from the CRC data. Hierarchical clustering of the CRC cohort was conducted using the “ConsensusClusterPlus” package. The distance between all possible pairs of clusters was calculated using the Euclidean distance formula, and the closest cluster pairs were merged into a new cluster.

$$Distance=\sqrt{\sum_{i=1}^{n}{\left(x_{i}-y_{i}\right)}^{2}}$$

Through this clustering analysis, the CRC samples were divided into two clusters. A consensus matrix was employed to determine whether the expression of genes related to septic shock is associated with CRC.

$$m_{ij}=\frac{\left(number\;of\;times\;samples\;i\;and\;j\;are\;in\;the\;same\;cluster\right)}{\left(number\;of\;resamplings\right)}$$

Subsequently, a heatmap was then used to explore the association between each cluster and SS expression patterns. The two clusters were subsequently categorized into SL-CR and NSL-CRC based on their association with SS.

### Survival analysis

Survival analysis of SL-CRC and NSL-CRC was performed using the “survival” package (Version 3.5–8) in R (Therneau, 2024), combined with clinical survival information obtained from TCGA (including observation days and survival status). The survival model was fitted using the using the survfit () function, employing the Kaplan-Meier method for estimating survival curves.

$$S\left(t\right)=\prod_{t_{i}\leq t}\left(1-\frac{d_{i}}{n_{i}}\right)$$

This approach evaluates the survival probabilities at different time points. Visualization of the survival analysis results was performed using the R package “survminer” (version 0.4.9). Additionally, the Cox proportional hazards model was fitted using the coxph function.

$$h\left(t\right)=h_{0\left(t\right)\exp}\left(\beta_{1}X_{1}+\beta_{2}X_{2}+\ldots +\beta_{p}X_{p}\right)$$

The summary function was used to extract the hazard ratios (HRs) and 95% confidence intervals from the Cox model, quantitatively describing the survival differences between subtypes. Finally, to further validate the survival differences, we conducted a non-parametric test using the survdiff function to assess survival time differences, providing additional statistical evidence for comparing survival times across different subtypes.

### Enrichment analysis

For enrichment analysis, differential expression analysis between SL-CRC and NSL-CRC subgroups within the CRC cohort was first conducted using the “limma” package (Version 3.56.2) to obtain log fold changes (with NSL-CRC as the control). Gene Set Enrichment Analysis (GSEA) was then performed on the CRC cohort using the “clusterProfiler” package (Version 4.8.3) (Wu et al., 2021), with a p-value <0.05 considered statistically significant for gene enrichment.

### Immune microenvironment analysis

To assess immune infiltration abundance, immune infiltration analysis was conducted on the CRC cohort gene expression matrix using ssGSEA algorithms, resulting in an immune cell infiltration matrix (Newman et al., 2019). Additionally, the ESTIMATE algorithm was used to evaluate stromal score, immune score, and ESTIMATE score for the CRC cohort.

### Immune therapy response evaluation

To evaluate and predict the response of CRC to immunotherapy, the Cancer Immunome Atlas (TCIA, https://tcia.at/) was used to predict the response of SL-CRC and NSL-CRC groups to anti-CTLA-4 and anti-PD-1 immune checkpoint inhibitors (ICIs). Furthermore, data on CRC tumor immune dysfunction and exclusion (TIDE) score, exclusion score, and dysfunction data were downloaded from the TIDE database (http://tide.dfci.harvard.edu/) to analyze immune escape and immune dysfunction in SL-CRC and NSL-CRC groups.

## Results

### Clinical information for CRC patients

The clinical information of all CRC patients is shown in Table 1 and Supplementary Table S1. A total of 598 CRC patients were included in the study, comprising 155 READ patients and 443 COAD patients. The majority of patients were of White race, followed by Black or African American; however, race was not reported for 45.81% of READ patients and 35.21% of COAD patients. Nearly all patients (99.35% and 99.55%) had primary tumors.

**TABLE 1:** Clinical information for CRC patients.

Variables	TCGA-READ (n = 155)	TCGA-COAD (n = 443)
Age	65.04 ± 11.22	66.98 ± 12.79
Gender		
Male	87 (56.13%)	238 (53.72%)
Female	68 (43.87%)	205 (46.28%)
Race		
Asian	1 (0.65%)	11 (2.48%)
White	77 (49.68%)	217 (48.98%)
Black or african american	6 (3.87%)	58 (13.09%)
Not reported	71 (45.81%)	156 (35.21%)
Pathological classification		
Primary	154 (99.35%)	441 (99.55%)
Recurrence	1 (0.65%)	1 (0.23%)
Metastatic	0	1 (0.23%)
History of colon polyps		
NO	49 (31.61%)	86 (19.41%)
YES	18 (11.61%)	92 (20.77%)
Not reported	88 (56.77%)	265 (59.82%)
Lymphatic invasion present		
NO	30 (19.35%)	82 (18.51%)
YES	33 (21.29%)	90 (20.32%)
Not reported	92 (59.35%)	271 (61.17%)

### Extraction co-expressed genes of SS-DEGs and TCGA-CRC

**FIGURE 1:**
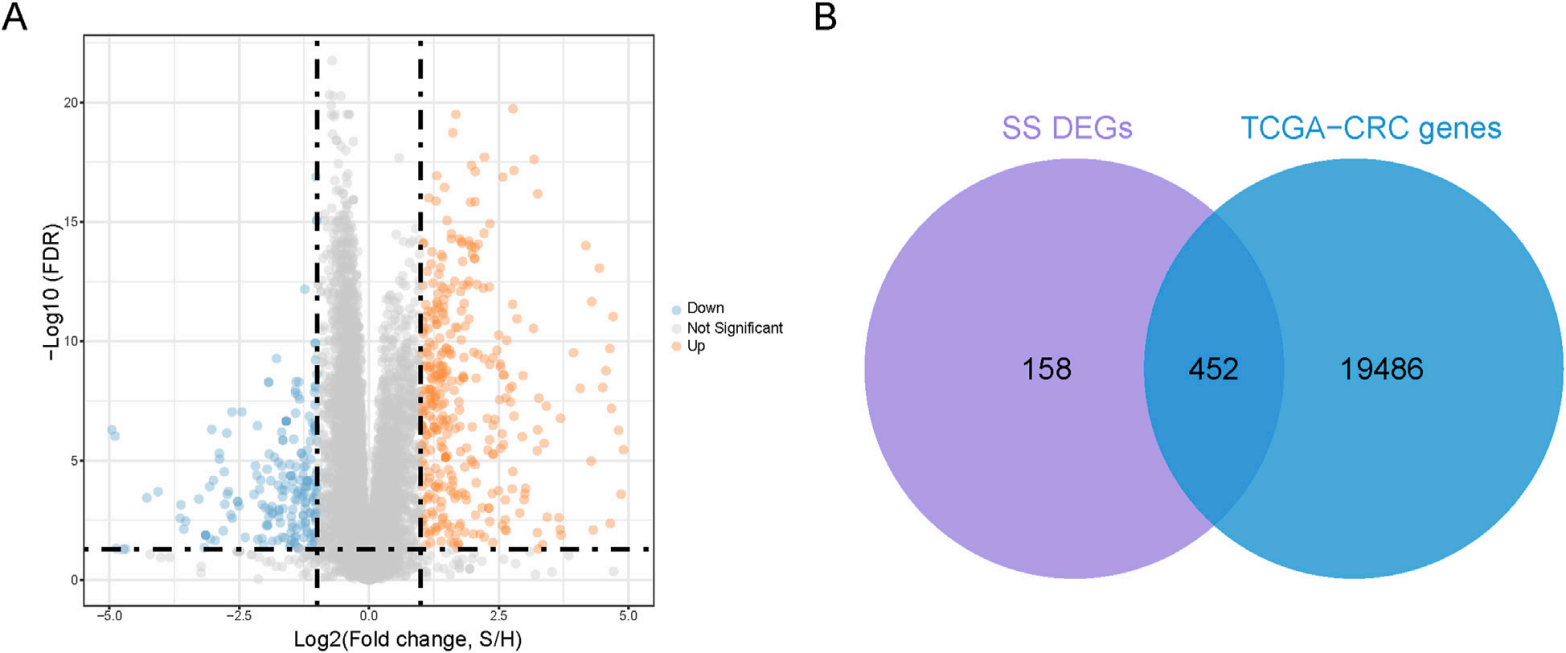
Identification of co-expressed genes. **(A)** Volcanic plot of SS-related differential expression genes (SS-DEGs) **(B)**. A Venn diagram is generated to represent 452 co-expressed genes of DEGs and TCGA-CRC genes.

We utilized the GSE26440 dataset with healthy participants as controls, employing an FDR <0.05 and |log2FC| >1 as the threshold for significant differential expression. This process identified 610 SS-DEGs, consisting of 387 upregulated and 223 downregulated genes (Figure 1A). Additionally, we intersected these 610 SS-DEGs with 19,938 genes expressed in CRC patients (TCGA-CRC genes), identifying 452 SS-DEGs expressed in CRC patients (Figure 1B).

### Hierarchical clustering associated with co-expressed genes

**FIGURE 2:**
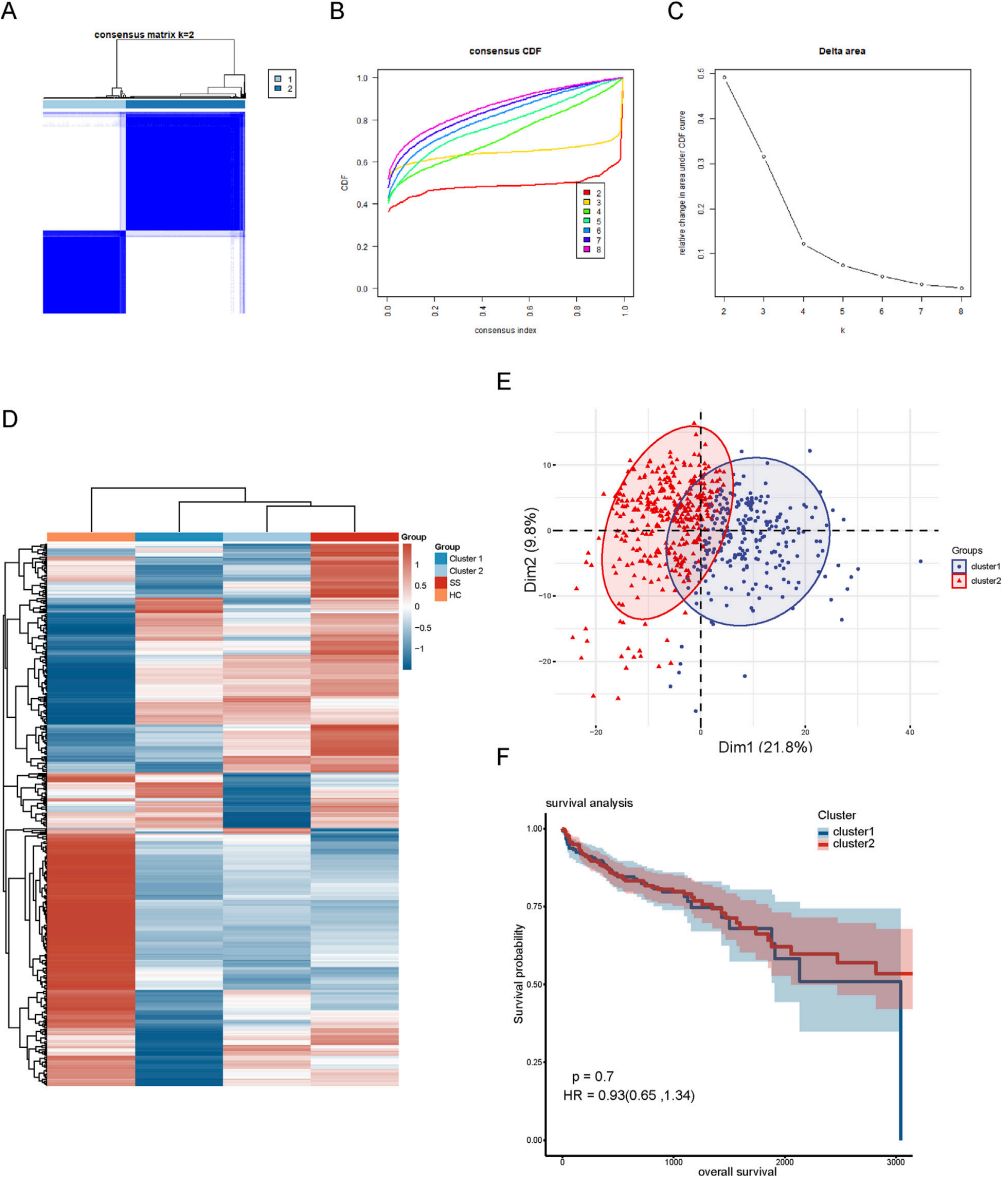
Hierarchical clustering based on 452 co-expressed genes. **(A)** Clustering heatmap of co-expressed genes. **(B)** Cumulative distribution function area of different clustering (k = 2–8). **(C)** The relative change of area under the CDF curve. **(D)** A heatmap of co-expressed genes in different clustering groups. **(E)** PCA plot of two clustering subgroups. **(F)** Analysis of overall survival between two cluster subgroups.

To explore the relationship of these 452 co-expressed genes with CRC subtypes, we conducted hierarchical clustering analysis. The cumulative distribution function (CDF) under different cluster numbers (2–8) is displayed in Figure 2B. The CDF curve was flattest at k = 2, indicating that a clustering into two groups yielded the most stable and consistent results. The relative change in the area under the CDF curve in Figure 2C was greatest at k = 2, after which the rate of change significantly diminished, suggesting that adding more clusters did not significantly improve clustering quality. Thus, we divided the CRC patients into two distinct clusters (Figure 2A). Additionally, we created a heatmap displaying the expression patterns of the 452 co-expressed genes in the two cluster subgroups. The results showed that the expression pattern of cluster 2 was similar to that of SS samples, leading us to designate cluster 2 as SL-CRC and cluster 1 as NSL-CRC. We further validated the independence between the two cluster subgroups using PCA, which showed significant separation (Figure 2E). However, the survival analysis revealed no significant difference in overall survival between the two cluster subgroups (Figure 2F).

### GSEA enrichment analysis of two cluster subgroups

**FIGURE 3:**
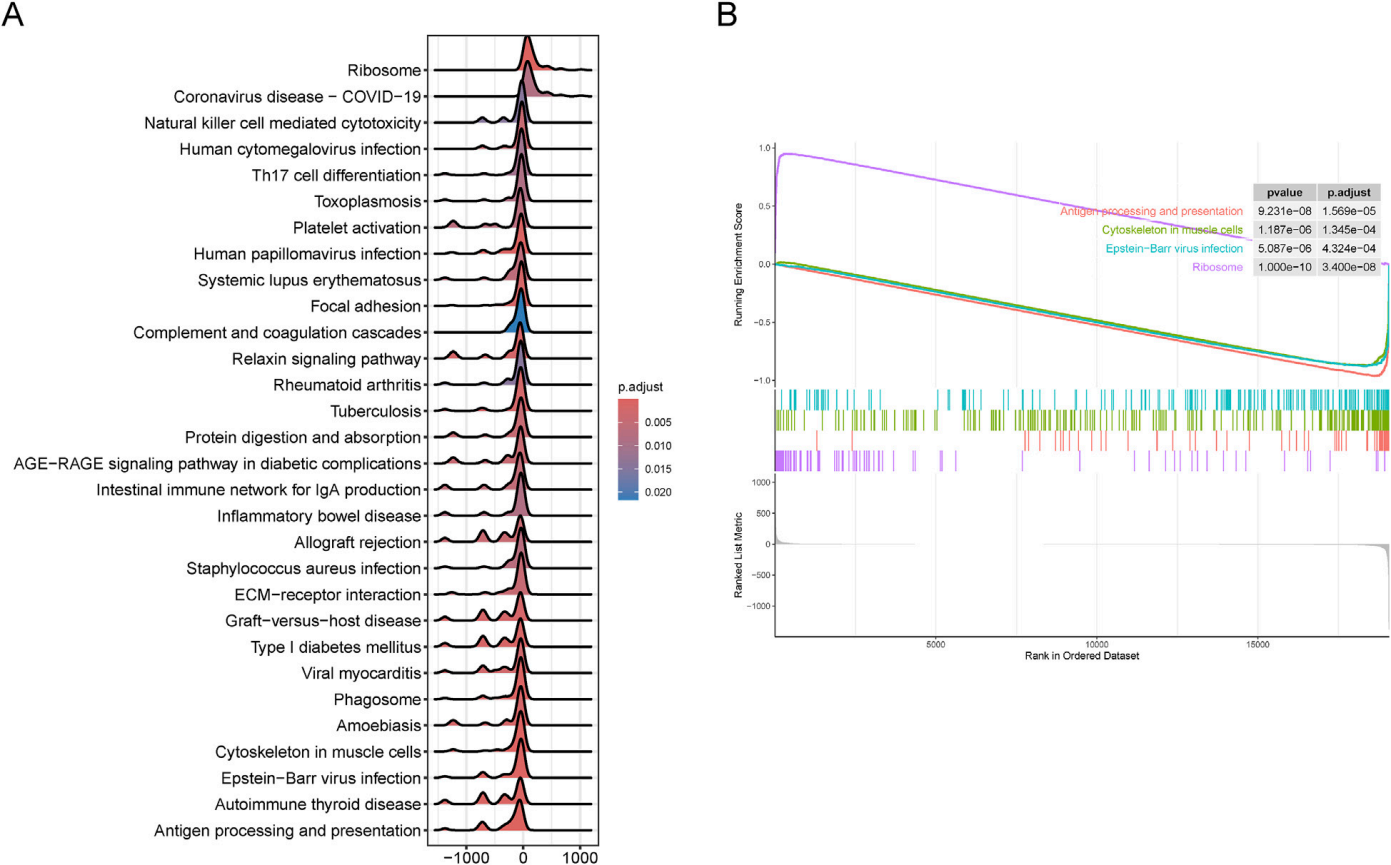
Gene set enrichment analysis (GSEA) between two cluster subgroups. **(A, B)** Visualization of representative results analyzed by GSEA. **(A)** Mountain plot. **(B)** Classic plot.

To further validate the clustering results related to SS, we identified DEGs between the SL-CRC and NSL-CRC subgroups. GSEA revealed that the DEGs between the two cluster subgroups are predominantly involved in pathways related to the ribosome, antigen processing and presentation, cytoskeleton in muscle cells, and Epstein-Barr virus infection (Figures 3A, B). The complete GSEA enrichment results are provided in Supplementary Table S2.

### Immune analysis of two cluster subgroups

**FIGURE 4:**
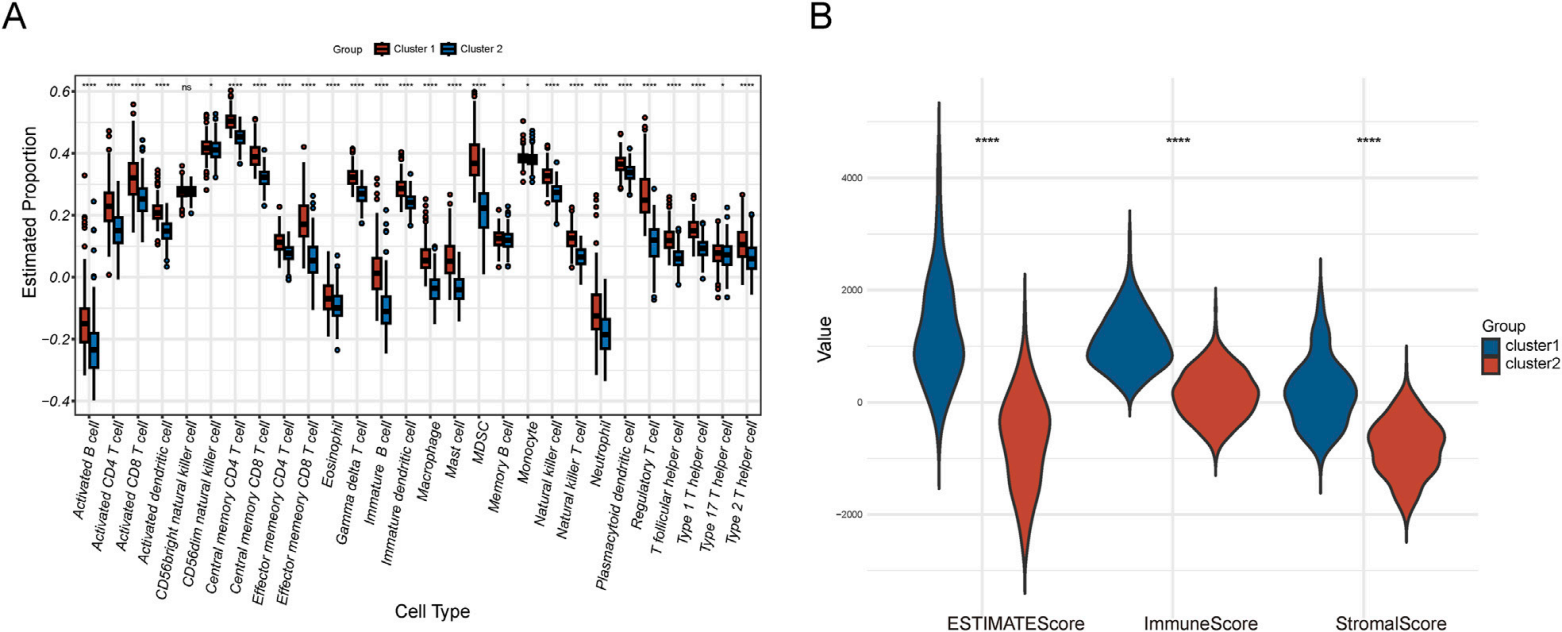
Immune microenvironment analysis. **(A)** Immune cell infiltration of the two clusters. **(B)** ESTIMATE score, immune score, and stromal score.

In the immune analysis of the two cluster subgroups, we utilized the ssGSEA algorithm to assess the infiltration levels of 28 immune cell types, identifying significant differences in 27 of these types (Figure 4A). Additionally, we employed the ESTIMATE algorithm to calculate the immune score, stromal score, and ESTIMATE score for the two subgroups. The immune score evaluates the proportion of immune cells, the stromal score assesses the stromal component proportion, and the ESTIMATE score reflects the overall non-tumorous cell component in the tumor microenvironment. The results showed that the ESTIMATE score, immune score and stromal score of the NSL-CRC subgroup were significantly higher than those of the SL-CRC subgroup (Figure 4B).

**FIGURE 5:**
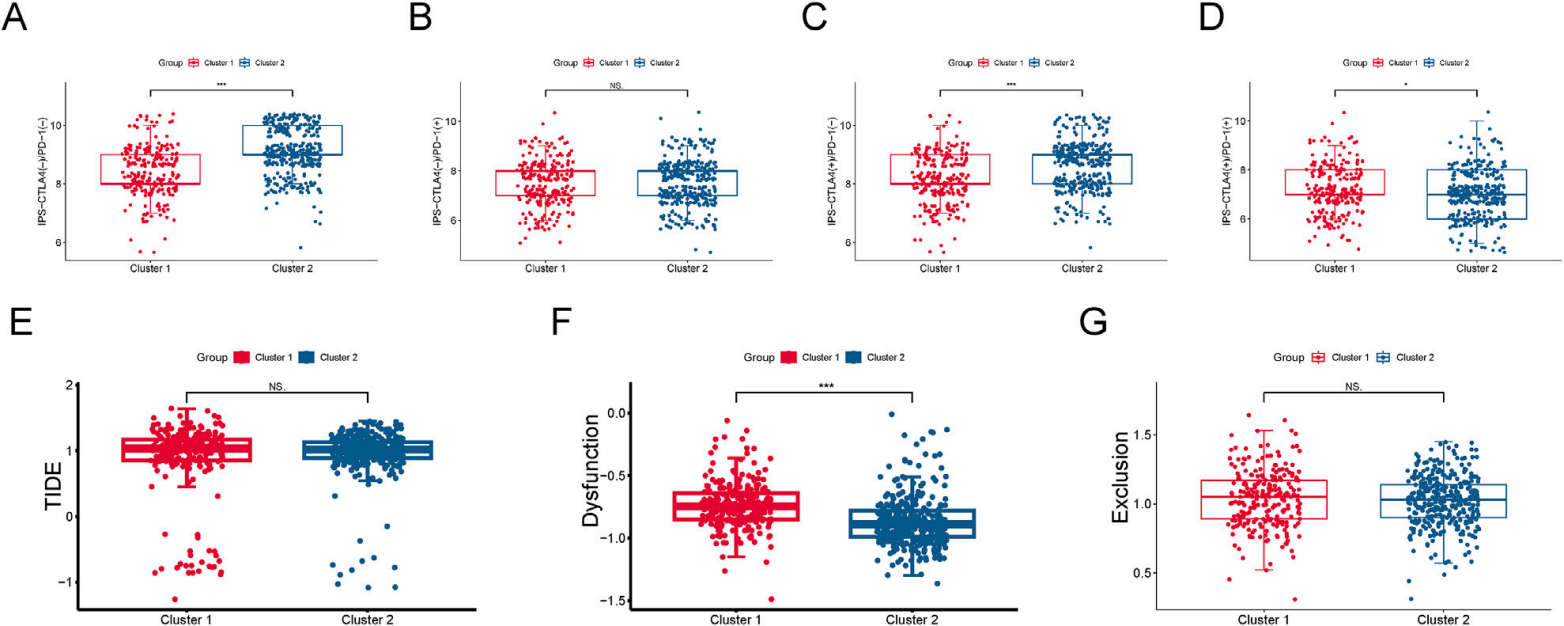
Immune therapy response evaluation. **(A–D)** Response of two cluster subgroups to anti-CTLA-4 and anti-PD-1 immune checkpoint inhibitors. **(E)** Dysfunction and exclusion (TIDE) score. **(F)** Dysfunction score. **(G)** Exclusion score.

Furthermore, based on the TCIA database, we predicted the differential responses of the two subgroups to anti-CTLA-4 and anti-PD-1 immune checkpoint therapies. In the absence of CTLA4 (−)/PD-1 (−) inhibitors, cluster 2 (SL-CRC) patients exhibited higher scores, suggesting they may have a stronger natural immune response or a more active immune state (Figure 5A). Cluster 2 patients showed higher response rates to CTLA4 (Figure 5C). Conversely, cluster 1 (NSL-CRC) might generate a more effective immune response when both CTLA4 and PD-1 ICIs are activated (Figure 5D). Additionally, using the TIDE database, we calculated the dysfunction score, exclusion score, and TIDE score for the two subgroups. The dysfunction score, which reflects factors in the tumor environment that hinder T cell immunity, indicated that cluster 1 might have more immune-suppressive factors in the tumor microenvironment, potentially reducing the responsiveness to immune therapy (Figure 5F). The exclusion score measures the difficulty of immune cells accessing tumor cells. The TIDE score, combining immune dysfunction and exclusion measures, did not show significant differences between the two cluster subgroups (Figures 5E, G).

## Discussion

Based on the expression of SS-DEGs in CRC patients, we classified CRC samples into two subtypes: SL-CRC and NSL-CRC. Although there was no statistically significant difference in overall survival between the two subtypes, they exhibited significant variations in the immune microenvironment and immune therapy responses, which could influence disease prognosis and treatment outcomes. With the decreasing cost and increasing acceptance of clinical sequencing technologies, these findings could play a role in stratifying CRC patients, aiding in the development of personalized treatment plans.

It is well known that the intestinal barrier is a crucial defense structure that prevents pathogens and toxins from entering the bloodstream. However, both sepsis and CRC can directly disrupt the structure of intestinal epithelial cells, increasing intestinal permeability. Cancer cells may also alter the intestinal microenvironment by secreting various pro-inflammatory and pro-angiogenic factors, impacting the integrity of the intestinal barrier (Gai et al., 2021; Sánchez-Alcoholado et al., 2020). Persistent intestinal infections and inflammation can lead to chronic stimulation and cell damage, potentially increasing the risk of CRC in the long term (Chen et al., 2024; Zuo et al., 2020). Sepsis, originating from pathogenic infections, progresses to shock driven by an uncontrolled immune response. Similarly, in cancers with an infectious origin, immune responses play a role in malignant transformation (Tripathi et al., 2020). GSEA confirmed that DEGs between the two CRC subtypes are involved in antigen processing and presentation, as well as Epstein-Barr virus infection, which are closely related to immune responses.

Our immune cell infiltration analysis identified significant differences across 27 immune cell types, with the majority showing higher infiltration levels in the NSL-CRC subtype compared to the SL-CRC subtype. This indicates that NSL-CRC may possess higher immune activity and a more complex immune response. The immune score, stromal score, and ESTIMATE score of NSL-CRC are all higher than those of SL-CRC, suggesting a more complex immune microenvironment potentially linked to tumor immune escape and tumor-promoting stromal remodeling activities (Yuan et al., 2023). The elevated immune cell infiltration and immune scores in NSL-CRC could theoretically indicate greater potential for responding to immune therapy. However, this could also reflect significant immune suppression, particularly in the presence of abundant immunosuppressive cells such as regulatory T cells (Sarkar et al., 2021; Tie et al., 2022). Despite NSL-CRC exhibiting higher immune cell infiltration and immune scores, its response to immune checkpoint inhibitors is not consistently optimal. SL-CRC shows higher treatment responses under CTLA4 (−)/PD-1 (−) and CTLA4 (+)/PD-1 (−) conditions, potentially due to fewer immunosuppressive factors or different immune regulatory mechanisms, allowing immune checkpoint inhibitors to more effectively “unleash” immune cells (Wang et al., 2021). These results suggest that combining therapies with different mechanisms may be more effective for subtypes like SL-CRC and NSL-CRC, which exhibit different immune therapy responses. For example, enhanced immunotherapies such as cancer vaccines or CTLA-4 immune checkpoint inhibitors might be more effective for SL-CRC (Jia et al., 2024). For SL-CRC, in addition to ICIs, a combination of therapies that modify the tumor microenvironment, such as matrix-degrading agents or anti-inflammatory treatments, might be necessary to improve immune cell infiltration and functionality (Beales, 2020; Najafi et al., 2019).

Our study, based on SS-DEGs, has preliminarily revealed the shared biological mechanisms between sepsis and colorectal cancer through the stratification of colorectal cancer patients, providing a foundation for further research. Of course, certain limitations should not be overlooked. First of all, our study relies on existing bioinformatics databases and publicly available datasets, which may limit the depth and breadth of our analysis. This limitation could be mitigated by integrating data from multiple sources or recruiting patients for sequencing studies. Secondly, although we identified potential roles for SS-DEGs in CRC, these SS-DEGs include genes that are directly related to the disease or are non-specifically expressed. Such a broad gene set may obscure crucial genes in differentiating sepsis-like and non-sepsis-like CRC, thereby limiting our understanding of these key biomarkers and potential therapeutic targets. Future studies should employ biological approaches such as weighted gene co-expression network analysis (WGCNA) and experimental validation to identify and confirm the key genes decisively influencing CRC subtypes. Thirdly, this study relies on publicly available databases, thus inherent biases such as ethnic bias and batch effects are inevitable. Of course, we processed the data to minimize the impact of batch effects. Finally, the TCGA-CRC samples, composed of combined TCGA-READ and TCGA-COAD parts, did not include other non-adenocarcinoma CRC samples such as squamous cell carcinoma. Currently, clinical research often treats colon cancer (CC) and rectal cancer (RC) as a single tumor entity of CRC. However, Paschke’s team highlighted differences between CC and RC across anatomy, epidemiology, molecular mechanisms, treatment responses, and prevention measures, advocating for abandoning the term CRC (Paschke et al., 2018). Therefore, merging these two datasets might obscure their differences. Future research may consider analyzing these diseases as separate entities to better understand their distinct biological behaviors.

We classified CRC patients into two subgroups: SL-CRC and NSL-CRC. Despite no significant differences in overall survival, we observed substantial variations in immune microenvironment and immune therapy responses between them. Our research provides a new perspective on understanding the biological diversity of CRC and lays the groundwork for identifying new therapeutic targets and developing personalized treatment strategies for specific colorectal cancer subtypes.

## Data Availability

The data presented in the study are deposited in the following repositories. CRC sample data are available in the TCGA repository (https://www.cancer.gov/tcga). SS sample data are available in the GEO repository, accession number GSE26440 (https://www.ncbi.nlm.nih.gov/geo/).

## Abbreviations

SS, Septic shock; CRC, Colorectal cancer; SL-CRC, SS-like CRC subgroups; NSL-CRC, non-SS-like CRC subgroups; GEO, Gene Expression Omnibus; GSEA, Gene Set Enrichment Analysis; ICIs, immune checkpoint inhibitors; TIDE, tumor immune dysfunction and exclusion; CDF, cumulative distribution function.
